# Genetic Evidence for Early Peritoneal Spreading in Pelvic High-Grade Serous Cancer

**DOI:** 10.3389/fonc.2018.00058

**Published:** 2018-03-07

**Authors:** Jeremy Chien, Lisa Neums, Alexis F. L. A. Powell, Michelle Torres, Kimberly R. Kalli, Francesco Multinu, Viji Shridhar, Andrea Mariani

**Affiliations:** ^1^Department of Internal Medicine, University of New Mexico Health Sciences Center, Albuquerque, NM, United States; ^2^Department of Biostatistics, University of Kansas Medical Center, Kansas City, MO, United States; ^3^Department of Biological Sciences, Emporia State University, Emporia, KS, United States; ^4^Department of Obstetrics and Gynecology, Mayo Clinic, Rochester, MN, United States; ^5^Division of Medical Oncology, Mayo Clinic, Rochester, MN, United States; ^6^Department of Laboratory Medicine and Pathology, Mayo Clinic, Rochester, MN, United States

**Keywords:** ovarian cancers, peritoneal spread, progression, cancer genomics, intratumor heterogeneity, phylogenetic analysis

## Abstract

**Background:**

Most pelvic high-grade serous (HGS) carcinomas have been proposed to arise from tubal primaries that progress rapidly to advanced disease. However, the temporal sequence of ovarian and peritoneal metastases is not well characterized.

**Methods:**

To establish the sequence of metastases, phylogenetic relationships among the ovarian and peritoneal carcinomas were determined from single-nucleotide variations (SNVs) in nine tumor regions from each patient with pelvic HGS carcinomas. Somatic SNVs from each tumor sample were used to reconstruct phylogenies of samples from each patient. Variant allele frequencies were used to reconstruct subclone phylogenies in each tumor sample.

**Results:**

We show that pelvic HGS carcinomas are highly heterogeneous, only sharing less than 4% of somatic SNVs among all nine carcinoma implants in one patient. TP53 mutations are found in all nine carcinoma implants in each patient. The phylogenetic analyses reveal that peritoneal metastases arose from early branching events that preceded branching events for ovarian carcinomas in some patients. Finally, subclone phylogenies indicate the presence of multiple subclones at each tumor implant and early tumor clones in peritoneal implants.

**Conclusion:**

The genetic evidence that peritoneal implants arose before or concurrently with ovarian implants is consistent with the emerging concept of the extra-ovarian origin of pelvic HGS cancer. Our results challenge the concept of stepwise spatial progression from the fallopian primary to ovarian carcinomas to peritoneal dissemination and suggest an alternative progression model where peritoneal spreading of early clones occurs before or in parallel with ovarian metastases.

## Introduction

The primary ovarian, peritoneal, and fallopian tube HGS carcinomas are inclusively categorized as the HGS carcinomas of the pelvis (1). Pelvic HGS carcinoma, the most common histological subtype of epithelial ovarian cancer, is also the most lethal form because it usually presents as a disseminated disease at the time of diagnosis (2). Although the ovary was previously considered as the primary for these carcinomas, emerging pathologic evidence suggests that the fallopian tube is the primary site for most pelvic HGS carcinomas (1). Precursor lesions are found in the fallopian tube in patients with pelvic HGS carcinomas (3, 4), supporting the fallopian tube as the primary site for these HGS carcinomas.

Following the initial spread to the ovary, single or clustered cancer cells detach from the ovarian carcinomas and seed to the peritoneal lining through passive transcoelomic spreading (5, 6). In addition, mathematical models based on clinical evidence suggest pelvic HGS carcinomas progress rapidly to disseminated disease (7). Therefore, pelvic HGS carcinomas may progress in a stepwise fashion from the primary lesions in the fallopian tube to the ovarian carcinomas to peritoneal metastases. Alternatives to the stepwise progression to peritoneal metastases are the model of concurrent progression to ovarian and peritoneal metastases from the tubal primary or the stepwise progression from peritoneal to ovarian metastases. However, the genetic evidence of whether ovarian carcinomas develop before peritoneal metastases is not yet available.

Metastasis is considered a clonal event because it is contributed by a tumor subclone that acquired metastatic potential (8). Therefore, mutations found in metastatic implants can facilitate the reconstruction of tumor subclones involved in metastatic processes (8). Moreover, by comparing the tumor subclones in different metastatic implants, it is possible to reconstruct the temporal pattern of metastatic events.

Recent advances in next-generation sequencing technologies now provide a better view of the spatial and temporal heterogeneity in the mutational landscape of chemotherapy-naïve and relapse HGS carcinomas (9). These studies suggest alterations in *TP53* to be the early genetic events (2, 10, 11), and these driver mutations are clonally dominant (12). Additional mutations, such as P3K3CA, CTNNB1, and NF1, can be found in a subset of tumor clones (subclones), and these mutations are considered branch driver mutations that facilitate branched evolution of tumor clones (12). Additional studies suggest the pattern of clonal evolution varies among patients (13–15). Hoogstraat et al. reported a high degree of shared mutations between primary tumor and peritoneal metastases in one patient, suggesting that metastases arose quickly from the primary carcinoma (13). Eckert et al. reported that peritoneal metastases can recolonize the tubal sites (15). Finally, Schwarz et al. (16) and McPherson et al. (17) reported the divergent pattern of clonal spreading suggestive of cross-seeding of subclones within peritoneal sites. Although these studies provide spatial and temporal heterogeneity of mutations and subclones and how these subclones contribute to intraperitoneal metastases, these studies have not addressed the critical question of whether ovarian carcinomas precede peritoneal carcinomas in the evolution of pelvic HGS cancer.

The sequence of metastases in pelvic HGS carcinomas has the potential to provide an answer to a long-standing challenge in the screening of early-stage HGS cancer. Previous screening results reported by the PLCO screening trial indicate that the most common screening approach that includes the annual CA-125 and transvaginal ultrasound was ineffective in detecting early-stage HGS cancer (18). Moreover, this approach produced an unacceptable rate of false positives leading to unnecessary surgeries and complications (18). Finally, this approach has not been shown to decrease disease-specific mortality (18). The fact that annual CA-125 and transvaginal ultrasound screening modalities are ineffective in detecting early-stage HGS cancer suggests these cancers progress to the advanced stage while they are in a low-volume disease that is below the limits of detection with available screening modalities. Finally, concurrent spreading of ovarian and peritoneal metastases or the peritoneal metastases that precede ovarian metastases may also explain the screening failures.

Whether or not pelvic HGS cancer progresses sequentially from the fallopian tube to the ovaries to the peritoneal sites is a clinically important question because it will affect how we can screen for early-stage pelvic HGS cancer. If the metastatic progression occurs sequentially through the ovarian sites, it may be possible to screen for cancer that is spatially confined to the pelvic regions. However, if metastasis does not follow the sequential progression through the ovarian sites but instead spreads to peritoneal sites at the same time or before ovarian metastases, it may be impossible to screen for pelvic HGS carcinomas that are confined to pelvic regions.

To gain insights into the progression of pelvic HGS carcinomas, we performed RNA sequencing of spatially distinct tumor samples from four patients. We included three ovarian tumors, three omental metastases, and three bowel metastases from each patient. We analyzed sequence variations from these tumor samples and identified somatic single-nucleotide variations (SNVs) from each tumor samples by subtracting germline SNVs found in the whole exome sequencing of matched normal blood. We then used somatic SNVs to reconstruct phylogenetic trees of tumor samples and tumor subclones within each tumor samples. These analyses provided genetic evidence that early-evolved tumor clones in pelvic HGS carcinoma contribute to peritoneal metastases.

## Results

### Common, Shared, and Unique Mutations in Tumor Samples

We analyzed somatic single-nucleotide variants (SNVs) from 36 spatially discrete regions of carcinomas from four patients with pelvic HGS cancer (Figure S1 and Tables S1 and S2 in Supplementary Material). Three ovarian sites, three metastases from the omentum, and three from the bowel were collected from each patient and subjected to RNA sequencing and SNV analysis. Since several methods for variant calling from RNA sequencing were available, we used three callers [SNPiR (19), RVboost (20), and MuTect2 (21)] to identify high-confidence variant calls made by all three callers (Figure S2A in Supplementary Material). In addition, we used the consensus calling from MuTect2 and GATK HaplotypeCaller (21) for the detection of small insertions and deletions (INDELs) (Figure S2B in Supplementary Material). SNPiR and RVboost were not used for calling INDELs because they were developed for the detection of SNVs. Because MuTect2 uses joint calling of variants from tumor RNA and normal DNA, the germline filter has no additional effect on consensus variant calls (hereafter referred to as Tier 1 variants, Figure S2C in Supplementary Material). To obtain variants with a higher degree of confidence, we further filtered out variants called at positions with coverage <5× in normal DNA and <10× coverage in tumor RNA. This approach further decreased the final list of somatic variants in each sample (Figure S2C in Supplementary Material). Finally, we filtered out germline variants reported in 1000 Genome Project. However, this filter had minimal effect on the remaining somatic variants (Figure S2C in Supplementary Material). The consensus calling method, that we named VaDiR (Variant Detection in RNA), produced high-confidence variant calls (22). After filtering out synonymous SNVs, we identified common and unique somatic variants within each anatomical region (Figure S2D in Supplementary Material). After applying the germline filters, coverage filters, and allele frequency filters (Figure S3 in Supplementary Material), each tumor sample contained a median of 98 SNVs (range, 60–117, Table 1).

**Table 1:** Filtered somatic single-nucleotide variations (SNVs) and insertions and deletions (INDELs) found in each patient.

	Ov1	Ov2	Ov3	Om1	Om2	Om3	Bw1	Bw2	Bw3
Patient 1	98	91	98	104	100	85	99	102	103
Patient 2	98	87	91	102	105	90	110	105	**117**
Patient 3	82	81	73	64	**60**	60	62	70	61
Patient 4	104	108	105	77	72	74	112	109	103

*SNVs detected by all three methods (RVboost, SNPiR, and MuTect2) and INDELs called by Haplotypecaller and MuTect2 were included. Germline SNVs (from exome sequencing of blood DNA from the same patient) were filtered out. In addition, for the variant positions where germline coverage is between 5 and 10×, variants from 1000 Genomes project were used to filter out potential germline variants. For SNVs, variants with VAF > 3% in normal DNA (because they are more likely to be germline variants) were filtered out. For INDELs, variants with VAF < 10% are filtered out. The total includes only those in coding, UTR, and ncRNA regions with at least 5× coverage in normal sample and 10× for each tumor sample. The median number of SNVs is 98 and range from 60 to 117. The workflow for the filter is shown in Figure S3 in Supplementary Material*.

**Figure 1:**
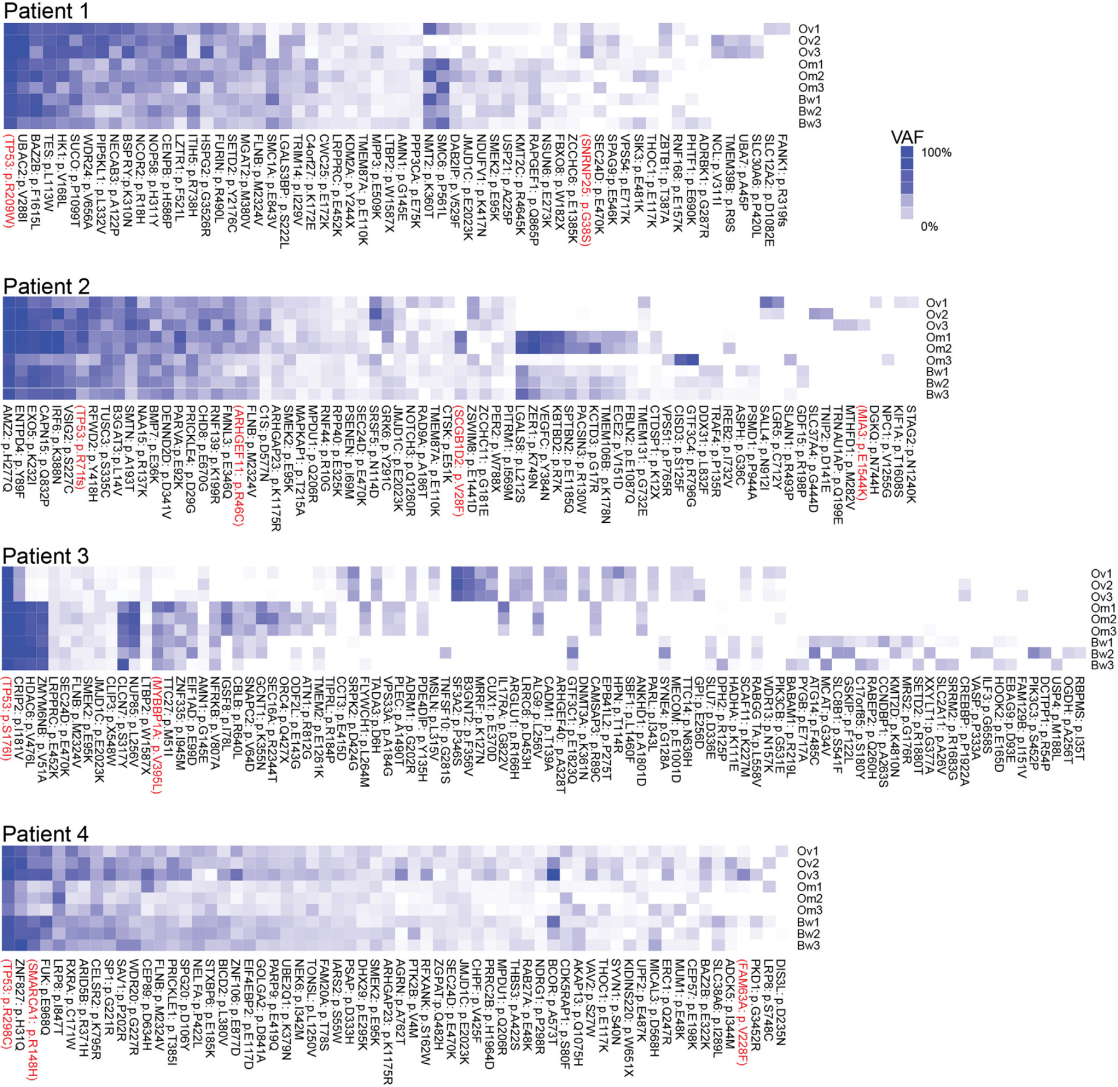
Multiregion sequencing of high-grade serous carcinomas identifies TP53 mutations as truncal mutations that are shared among all regions in each patient. Shared and unique mutations found in nine carcinoma samples in each patient are shown as mutation heatmaps. Truncal *TP53* mutations, identified in all patients, were also reported in COSMIC and detected at high variant allele fractions in all but one patient. Interestingly, these patients also harbor truncal single-nucleotide variations (SNVs) in epigenetic regulators, such as SETD2 (Patient 1), CHD8 (Patient 2), HDAC6 (Patient 3), and SMARCA1 and ARID5B (Patient 4). We also found SNVs that are shared only within the ovarian sites or within metastases. SNVs recorded in the COSMIC database are shown in red. Variant allele frequencies (VAFs) for each detected mutation are color coded. Dark blue = 100% VAF; white = 0% VAF.

Next, we assessed intratumor heterogeneity by determining shared and unique mutations in nine carcinomas from each patient. Differences in coverage at any given region in all tumor samples from each patient would potentially produce false-positive unique mutations and bias the assessment of intratumor heterogeneity. For example, among nine samples from each patient, if a variant is found in one sample with at least 10× coverage at the position but no variant is detected in other samples where coverage at the position is less than 5×, it would be difficult to ascertain if such variant is unique to one sample alone because not enough information is available for the remaining eight samples. Therefore, to accurately assess intratumor heterogeneity, we only considered variant positions where we had at least 5× coverage in normal DNA and at least 10× coverage in all tumor RNA from each patient. A modest number (15–60%) of SNVs were shared within implants from a single anatomic region from a particular patient, and an even smaller fraction of SNVs (2–25%) were shared among all nine sites in any patient, suggesting a high degree of intratumor heterogeneity (Table 2). Truncal mutations, such as *TP53* mutations, were shared among all sites and observed at high allele frequencies (Figure 1; Tables S3–S6 in Supplementary Material). Visual inspection of sequence files from each patient showed high-frequency sequence variations in *TP53* in Patients 1 (R248W), 3 (S215I), and 4 (R337C) (Figures S4A–C in Supplementary Material). Carcinoma samples in Patient 2 contained a single-base frameshift deletion (R110fs*13) that likely caused non-sense-mediated decay because the *TP53* transcript was not as abundant as in other samples (Figure S4D in Supplementary Material). In addition, mutations in genes associated with epigenetic regulators, such as *SETD2, CHD8, HDAC6*, and *SMARCA1* were observed in these patients (Figure 1; Figures S5–S8 in Supplementary Material).

**Table 2:** Total and shared single-nucleotide variations (SNVs) in specific regions and all regions.

	Patient 1		Patient 2		Patient 3		Patient 4	
	Mutations	Shared (%)	Mutations	Shared (%)	Mutations	Shared (%)	Mutations	Shared (%)
Ovarian	36	17 (47.22)	47	24 (51.06)	32	14 (43.75)	50	17 (34.00)
Omentum	40	24 (**60.00**)	51	21 (41.18)	34	19 (55.88)	18	4 (22.22)
Bowel	39	22 (56.41)	46	26 (45.52)	46	7 (**15.22**)	48	22 (45.83)
All regions	59	13 (22.03)	76	19 (**25.00**)	94	2 (**2.13**)	62	4 (6.45)

*Shared SNVs at all regions varied from 2.13 to 25%. Within each anatomical region (ovarian, omental, or bowel), shared SNVs varied from 15.22% (bowel metastasis) to 60% (omentum). In addition to the filtering steps in Table 1, additional filters included: no synonymous variants, no variants in UTR nor ncRNA, and only positions with read depth ≥5 in normal DNA and ≥10 in all tumor RNA are considered*.

### Phylogeny of Tumor Samples in Each Patient

**Figure 2:**
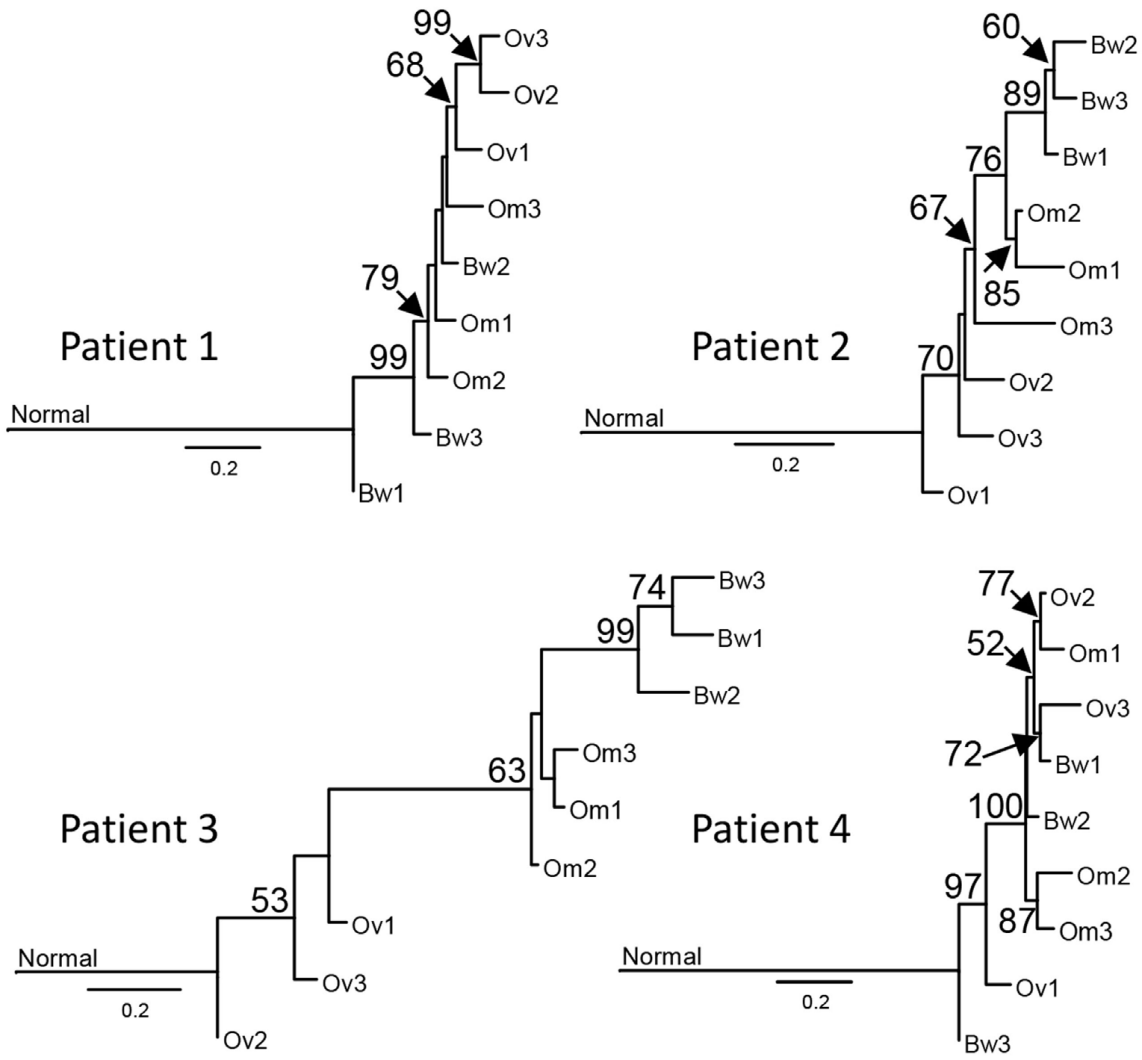
Sample phylogenies indicate that intraperitoneal carcinomas descended from ancestral cancer that existed before the diversification of ovarian carcinomas. Somatic single-nucleotide variations were used for maximum likelihood analysis with GARLI 2.0 (24) to construct phylogenetic trees for each sample. Best trees representing the sample phylogenies for four patients are shown. The numbers at the branches are confidence values based on the bootstrap method. Results indicate that tumor lineages found in the bowel arose before the diversification of ovarian tumor lineages in Patients 1 and 4. The scale bar indicates the number of substitutions per site; branch lengths are proportional to amounts of mutational change. For purposes of presentation, the genetic distance between normal cells and the base of the cancer radiation is shown one-third scale. A majority of mutations in all patients, except Patient 3, are assigned to the ancestral tumor clone before the emergence of all tumors, consistent with the results from prior studies (23).

Next, we used synonymous and non-synonymous somatic SNVs to perform phylogenetic analyses of nine carcinoma samples from each patient. Majority rule consensus evolutionary trees for nine tumor samples indicate that some intraperitoneal carcinomas (in Patients 1 and 4) descended from ancestral cancer that existed before the common ancestor of the ovarian carcinoma samples (Figure 2). The distance from the base of the tree is proportional to the number of mutations that are different from the normal sample. The results indicate the majority of mutations are assigned to ancestral tumor clone before the emergence of all tumors (Figure 2). These results are consistent with a recent study by Tomasetti et al. that indicated that a majority of somatic mutations in cancer originate before tumor initiation (23).

### Phylogeny of Tumor Subclones in Each Sample

**Figure 3:**
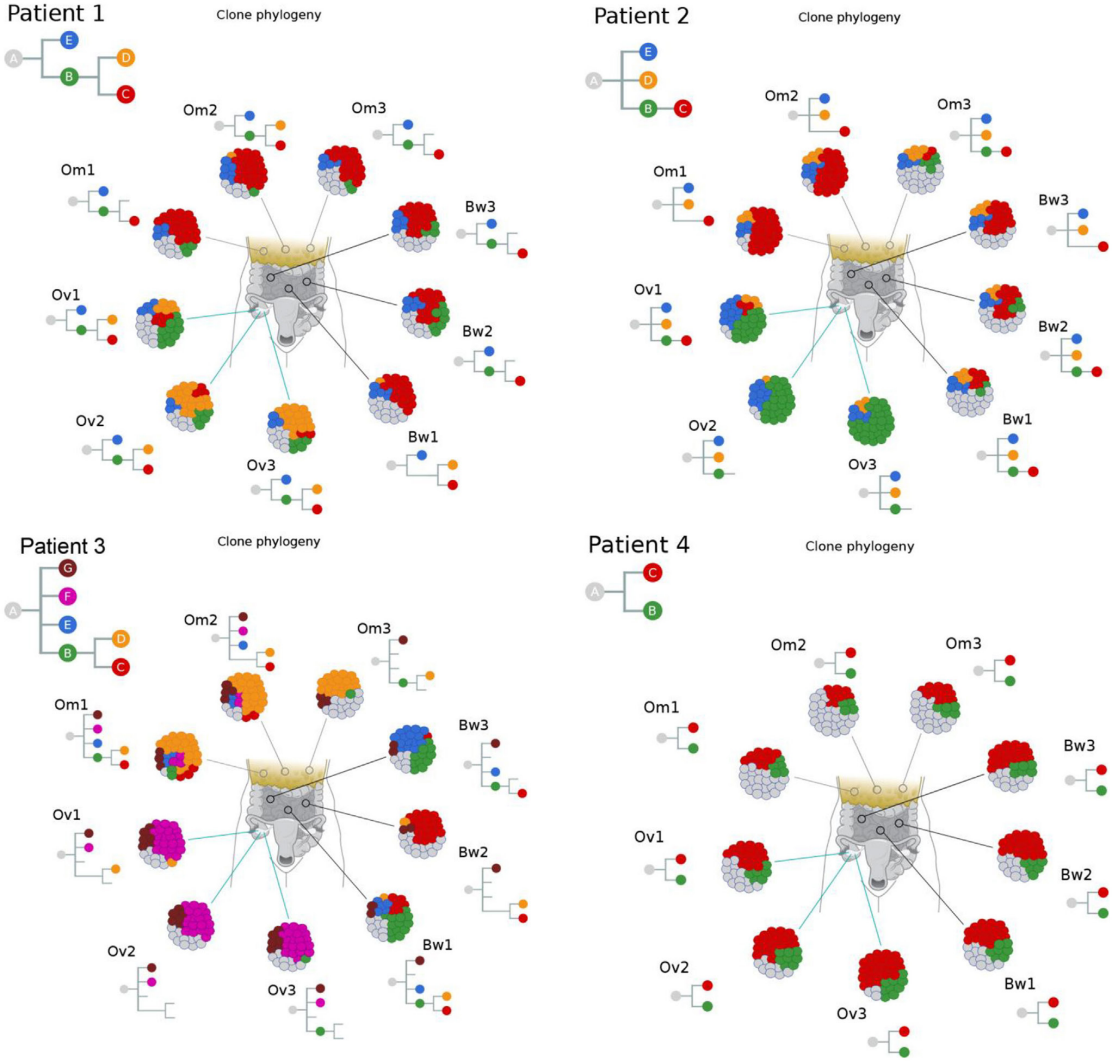
Clonal phylogenies indicate the presence of ancestral tumor clones at intraperitoneal implants. Clonal inference analysis indicates the presence of multiple clones at each tumor site. The clonal hierarchy and prevalence at nine tumor implants are shown in the schematic tumor and clones with distinct genotypes are color coded. Hierarchical relationships between clones are shown as inverted trees where normal cells are represented as clone “A.” These results indicate the presence of ancestral tumor clones (direct progenies from clone “A”) in various peritoneal implants in all four patients.

We next used Clonal Inference of Tumors Using Phylogeny (CITUP) bioinformatic program which analyzes somatic variant allele frequencies (VAFs) to infer cancer subclones in each carcinoma implants. The results indicate the presence of multiple tumor subclones at each tumor implants as well as “ancestral” early-evolved cancer clones in intraperitoneal metastatic sites in each patient (green “B” clones in Figure 3). A similar observation can also be made from a recent study by McPherson et al. that showed early-evolved cancer clones in several intraperitoneal metastatic sites in all seven patients (17).

## Discussion

In this study, we performed RNA-seq analysis of nine tumor samples from each patient with pelvic HGS carcinomas. In each patient, all nine tumor samples from ovarian, omental, and bowel tumors contain shared mutations in *TP53*. All these mutations are previously reported as somatic mutations in the COSMIC database (25). These results suggest *TP53* mutations are ancestral genetic events that are inherited in all descendant tumor clones that spread to various tumor sites in the ovaries and the peritoneum. These results are consistent with previous studies indicating that *TP53* is frequently mutated in pelvic HGS cancer (2, 10, 11). In addition to *TP53* mutations, mutations in genes associated with epigenetic regulators, such as SETD2, CHD8, HDAC6, and SMARCA1 are also observed in these patients. Mutations in these genes are emerging as pathologically relevant somatic mutations in various cancer types (26–30).

Our results from the phylogenetic analysis of sequence variations in tumor samples suggest some intraperitoneal carcinomas (in Patients 1 and 4) descended from ancestral cancer that existed before the common ancestor of the ovarian carcinoma samples. These results are consistent with results from recent studies by McPherson et al. (17) and Schwarz et al. (16), indicating peritoneal carcinomas with early branching events that preceded the branching events for ovarian carcinomas. Moreover, another study by Lee et al. included one case with ovarian carcinomas and several metastases (14). Lee et al. produced two phylogenetic trees using validated somatic mutations and copy number alterations. In their trees, peritoneal metastases are derived from lineages that preceded or that are sister to the common ancestors of genetically divergent ovarian carcinomas (14). These results further support the parallel progression of ovarian and intraperitoneal carcinomas in pelvic HGS cancer. The concurrent or parallel growth of ovarian and peritoneal carcinomas may present as a disseminated disease at the time of diagnosis, resulting in a high rate of advanced disease for pelvic HGS cancer (2).

**Figure 4:**
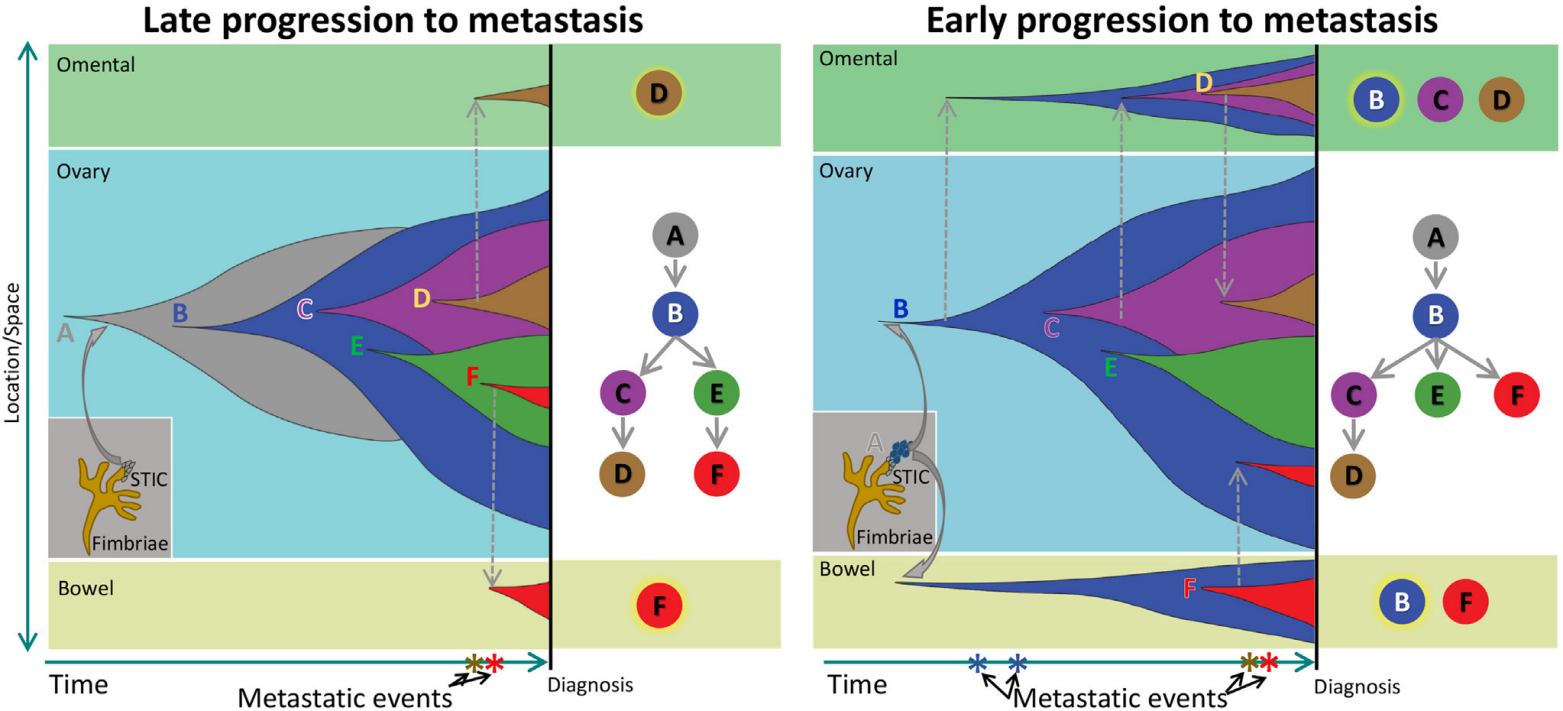
Schematic of clonal progression in cancer. A normal cell acquires genetic alterations that enhance the growth “fitness” of its descendants to produce carcinoma *in situ* (CIS). A CIS clone acquires additional mutations that enable escape from the confines of the hypoxic microenvironment in the primary niche, producing diffuse cancer. In the Late Progression model, this ancestral clone acquires additional clonal driver mutations, and ultimately, a descendant late in the evolutionary sequence acquires metastatic properties and colonizes a metastatic niche. In the Early Progression model, the ancestral clone colonizes a metastatic niche and progresses in parallel to the ancestral clone in the primary niche.

Our results from the clonal phylogenetic analysis suggest the presence of early-evolved tumor clones in omental and bowel metastases. This result is significant because it suggests early-evolved tumor clones have metastatic properties and propensity to colonize the peritoneum. In a review, Greaves and Maley described clonal evolution in cancer that follows a stepwise progression to metastasis (8). In their model, metastases are produced by late-evolved clones that acquired metastatic potential through accumulated genetic changes, and this model can be considered as late divergent progression to metastasis (Figure 4). However, cancer clone can also acquire metastatic potential early in the divergent evolution, and this model can be considered as early divergent progression to metastasis (Figure 4). The existence of early or late divergent progression to metastasis can be inferred from the analysis of tumor subclones. If metastases are established through late divergent evolution, late-evolved tumor clones that acquired metastatic potentials are expected to inhabit the metastatic sites (Figure 4). On the other hand, if metastases are established through early divergent evolution, early-evolved tumor clones (for example, clone “B”) should exist in metastases (Figure 4). Results from our study as well as McPherson et al. indicate the presence of early-evolved tumor clones in intraperitoneal metastases (17), thereby providing the first phylogenetic evidence of early divergent tumor clones contributing to metastases in pelvic HGS cancer.

It should be noted that our current study is limited to four cases out of 43 patients. These four patients were selected because nine tumor samples (three ovarian, three bowel, and three omental tumor samples) were available from these patients. Other patients were excluded from the study because they do not have three frozen tumor samples from each anatomical site. However, future studies will focus on including additional patients as well as tubal primary tumors if they are available.

Our study has additional limitations. First, we used VAFs from RNA sequencing instead of relying on more robust DNA sequencing from the exome. Due to allele-specific gene expression, VAFs at DNA and RNA levels may not be correlated. While developing the method to detect high-confident sequence variations from RNA sequencing, which we named VaDiR, we used data sets from the Cancer Genome Atlas which contain both RNA sequencing and DNA exome sequencing. The results from these analyses indicate that VAFs at RNA and DNA levels are highly correlated (22). Another concern is that RNA editing could confound the variant discovery from RNA sequencing. To remove this confounding factor, we filtered out all known RNA editing sites and did not consider variants at these positions.

Another limitation of our study is the lack of primary tumor samples. To overcome this limitation, our analysis focused on the phylogenetic relationship among three tumor sites (ovarian, omental, and bowel) relative to the normal sample. Although primary tumor samples are missing in the phylogenetic trees, the emergence of metastatic tumor samples can be inferred with respect to normal samples. Future studies should include primary tumor samples within the fallopian tube. However, such studies should be cognizant of the fact that primary tumors may contain metastases from peritoneal implants as previously reported (15), and it may be difficult to perform phylogenetic analysis without properly microdissecting the tubal primary site.

Another concern is that copy number alterations, that are characteristics of HGS, could produce inaccurate measures of VAFs and that these inaccuracies will affect clonal inferences. Although we acknowledge this limitation, our overall conclusion that early-evolved clones are observed in peritoneal metastases is based not solely on our results but also on the results produced from single-cell DNA sequencing reported by McPherson et al. (17). In addition, the phylogeny of tumor samples, which is less affected by discordances in VAFs between RNA and DNA because the analysis uses binary information (variant vs non-variant positions), indicates early divergence of peritoneal metastases that often precede the divergence of ovarian carcinomas. Therefore, multiple lines of evidence from independent studies supports the main conclusion of this study that peritoneal metastases occur early in the clonal evolution, and that peritoneal metastases can often precede ovarian carcinomas in pelvic HGS cancer.

The propensity for pelvic HGS cancer to spread to distant sites at early stages of cancer progression is consistent with the tubal origin of these cancers, in which precursor lesions in the tube may seed to the ovary as well as to the peritoneum. Concurrent growth of ovarian and peritoneal tumors may present as a disseminated disease at the time of diagnosis, resulting in a high rate of advanced disease for pelvic HGS cancer. A recent study by Schwarz et al. included two cases with ovarian carcinomas in their multiregion sequencing analysis (16). Although the focus of their study was a phylogenetic analysis of spatial and temporal heterogeneity in HGS cancer, phylogenetic trees placed ovarian carcinomas within the clade with omental or bowel metastases, and some omental metastases showed early branching events that preceded the branching events for ovarian carcinomas. Moreover, another study by Lee et al. included one case with ovarian carcinomas and several metastases (14). Lee et al. produced two phylogenetic trees using validated somatic mutations and copy number alterations. In both trees, peritoneal metastases either preceded or occurred concurrently with ovarian carcinomas (14). These results are consistent with our findings and the model of tubal carcinogenesis that can often progress concurrently to ovarian and peritoneal metastases.

These findings have important implications for the efforts at early detection of pelvic HGS cancer because the window for early detection is predicted to be short, and the disease volume may be minimal at the beginning of dissemination. Moreover, a concurrent dissemination of HGS carcinomas from tubal sites to the ovarian and peritoneal sites would undermine current screening modalities that focus on detection of adnexal masses. Consistent with this notion, recent large-scale screening by Menon et al. identified several women with ovarian cancer who displayed a rapid rise in CA-125 within a few months between screenings, and the majority of these women were diagnosed at advanced stages (31). Therefore, future efforts to improve sensitivity and specificity of detecting early-stage pelvic HGS cancer should include screening for extra-ovarian precursor lesions with genetic biomarkers to supplement existing protein-based biomarkers.

## Materials and Methods

### Patient Material

This study was approved by the Mayo Clinic institutional review boards, and the samples were obtained from the Division of Gynecology Oncology at Mayo Clinic, Rochester, MN, USA. From a population of 43 patients who consented to the study (between 08/01/2010 and 07/31/2011) and underwent surgery for ovarian cancer, we selected 4 cases based on the following characteristics: each of the patients had three separate fresh frozen cancer tissues from each one of 3 different sites (ovary, bowel, and omentum). Sufficient quality and quantity of the RNA were obtained from the samples. Three patients, included in the study, were diagnosed with HGS ovarian cancer stage IIIC/IV and one patient with primary peritoneal serous cancer. From each patient, we analyzed three regions from the ovarian tumor, three regions from the omentum metastasis, and three regions from bowel metastasis (Table S1 in Supplementary Material).

Ovarian carcinomas were collected from the adnexal region and include the ovary and possibly the tube. Omental metastases were taken from the omentum, and bowel metastases were taken from metastases to the large bowel. For the one patient with primary peritoneal carcinoma, the “primary tumor,” was collected from the cul-de-sac.

All four patients underwent debulking surgery and complete surgical staging at Mayo Clinic, and none of them received any chemotherapy before surgery. WHO criteria were used to evaluate the histologic subtype and grade of the tumors.

The tissue was obtained through the following process: samples varying from 0.5 to 3 cm were collected by the surgeon intraoperatively and snap frozen in liquid nitrogen within 30 min after surgery. From each sample, a hematoxylin and eosin staining slide was reviewed by an experienced pathologist. Only samples with more than 70% of cancer cell were selected for total RNA extraction. Two hematoxylin and eosin staining control slides were obtained at the beginning and at the end of the cutting sections for the RNA extraction. All tumors had between 5 and 10 mm side length, and four 10 µm sections of each sample were used for RNA extraction.

### Total RNA Extraction

Total RNA was extracted from 36 samples with Qiagen reagent. The RNA samples were quantified using a Nanodrop Spectrophotometer and qualified using the Agilent Bioanalyzer. The majority of samples (34 out of 36) used in the study had RIN above 7.0 (Table S2 in Supplementary Material) and were kept at −80°C after purification and after qualification and quantification.

### mRNA Library Preparation and Sequencing

RNA libraries were prepared according to the manufacturer’s instructions for the TruSeq RNA Sample Prep Kit (Illumina, San Diego, CA, USA). The concentration and size distribution of the libraries were determined on an Agilent Bioanalyzer DNA 1000 chip (Santa Clara, CA, USA). Libraries were loaded onto flow cells at concentrations of 8–10 pM to generate cluster densities of 700,000/mm^2^ following Illumina’s standard protocol using the Illumina cBot and cBot Paired End cluster kit version 3. The flow cells were sequenced as 51 × 2 Paired End reads on an Illumina HiSeq 2000 using TruSeq SBS sequencing kit version 3 and SCS version 1.4.8 data collection software. Base calling was performed using Illumina’s RTA version 1.12.4.2. There were approximately 45 million reads per sample mapped to the human genome, and 21,686 genes were detected.

### SNV Detection

Single-nucleotide variations from RNA sequencing were identified using VaDiR, a method that we have recently developed (22). Briefly, paired-end reads from sequencing were mapped to the hg19 human reference genome using the STAR aligner (32). SNVs were detected from RNA sequencing datasets using RVboost (20), SNPiR (19), MuTecT2 (21), and GATK Best Practice for variant calling on RNAseq (33). Resulting VCF files were processed with bcftools to identify variants called by at least three methods. VCF files from each patient were merged with bcftools, filtered by germline variants from normal blood samples of the patient, and annotated with ANNOVAR (34). Samtools mpileup was used to obtain read depth. Only those variants with at least 10× coverage in each of tumor samples and 5× coverage in the normal sample were accepted. Variants found in 1000 genome SNVs (except where no variant was detected at the position with at least 10× coverage in the normal genome) were also filtered out. Synonymous and non-synonymous SNVs and INDELs from at least two callers were used to generate the tumor phylogeny for each patient.

### Phylogenetic Tree Analysis

All somatic SNVs were concatenated and treated as sequence alignments in phylogenetic analyses. The best substitution model for each dataset (SNVs from a single patient) was selected with the BIC in jModelTest 2 (35). Relationships among clones within patients were inferred under maximum likelihood (ML) using GARLI 2.0 (24). Heuristic searches for the best ML tree for each dataset were conducted from 100 random starting points (i.e., searchreps), and nodal support was evaluated with 200 bootstrap replicates, each from a single random starting point. Majority rule consensus trees with support values were produced from the GARLI bootstrap trees using PAUP*4.0a149 (36), and then the support values were mapped onto the single best ML tree topology for presentation.

### Clonal Inference in Multiple Tumor Samples Using Phylogeny

Variant allele frequencies for filtered somatic non-synonymous SNVs from three callers were used for phylogenetic tree analysis using the CITUP bioinformatics tool (37). Iterative method called CITUP_iter, included in the CITUP program, was used to generate trees with up to nine nodes. The optimal trees and proportional representation of each tumor clone, given by CITUP, were illustrated for each patient using Inkscape.

### Visualization of Shared and Unique SNVs from Each Patient

Non-synonymous somatic SNVs that were detected in each patient were plotted as heatmap that is color coded with a gradient of blue representing VAF using heatmap.2 from gplots in R. SNVs that match documented somatic variants in the COSMIC database are highlighted in red labels.

Scripts used to process data sets can be found at the following public link. https://osf.io/e2z7y/.

## Data and Materials Availability

All data used in this study are publicly available for review at the following link. https://osf.io/awby2/.

## Ethics Statement

This study was approved by Mayo Clinic Institutional Review Board.

## Author Contributions

JC, LN, AP, VS, AM, and MT prepared the manuscript. JC, AM, MT, VS, and KK designed the research studies. MT and FM processed samples. JC and LN analyzed the data and prepared the data visualization. AP carried out analyses of phylogenetic relationships among tumor samples. FM reviewed pathology.

## Conflict of Interest Statement

The authors declare that the research was conducted in the absence of any commercial or financial relationships that could be construed as a potential conflict of interest.
