# Supplementary material for: Genetic Evidence for Early Peritoneal Spreading in Pelvic High-Grade Serous Cancer
Source: Front Oncol. 2018 Mar 7;8:58. doi: 10.3389/fonc.2018.00058 (PMC5858520; doi:10.3389/fonc.2018.00058)
Supplement: Supplementary file 1 [file Data_Sheet_1.PDF]

Supplementary Materials

Table S1. Clinicopathologic characteristics of patients and samples included in the study

Patient	Age	Cancer Type	Tumor Grade	Tumor Histology	Tumor Stage	Regions	Total number of specimens
1	76	Primary Ovarian	High	Serous	IIIC	Primary Ovary	3 (Ov1,Ov2,Ov3)
						Omentum	3 (Om1,Om2,Om3)
						Bowel	3 (Bw1,Bw2,Bw3)
2	63	Primary Ovarian	High	Serous	IIIC	Primary Ovary	3 (Ov1,Ov2,Ov3)
						Omentum	3 (Om1,Om2,Om3)
						Bowel	3 (Bw1,Bw2,Bw3)
3	65	Primary Ovarian	High	Serous	IIIC	Primary Ovary	3 (Ov1,Ov2,Ov3)
						Omentum	3 (Om1,Om2,Om3)
						Bowel	3 (Bw1,Bw2,Bw3)
4	68	Primary Peritoneal	High	Serous	IV	Primary (cul-du-sac)	3 (Ov1,Ov2,Ov3)
						Omentum	3 (Om1,Om2,Om3)
						Bowel	3 (Bw1,Bw2,Bw3)

Table S2. RNA integrity number (RIN) for samples used in the study.

	Patient 1	Patient 2	Patient 3	Patient 4
**Regions**		**RIN**		
Ovarian (Ov1)	7.2	8.1	7.1	8.9
Ovarian (Ov2)	7.4	8.2	7.8	8.2
Ovarian (Ov3)	7.1	8.4	7.5	7.5
Bowel (Bw1)	7.2	8.3	7.7	7.2
Bowel (Bw2)	7.5	7.3	7.8	8.1
Bowel (Bw3)	7.4	7.7	6.8	7.9
Omentum (Om1)	7.8	8.3	7.5	8.8
Omentum (Om2)	6.7	8.1	7.1	9.5
Omentum (Om3)	7.2	8.1	7.7	8.2

RIN > 7 are considered sufficient quality for RNA sequencing studies.

Large File - **Table S3.** SNVs detected in Patient 1. This table can be downloaded from the following public link. https://osf.io/e2z7y/

Large File - **Table S4**. SNVs detected in Patient 2. This table can be downloaded from the following public link. https://osf.io/tbvrp/

Large File - **Table S5**. SNVs detected in Patient 3. This table can be downloaded from the following public link. https://osf.io/5r4gd/

Large File - **Table S6**. SNVs detected in Patient 4. This table can be downloaded from the following public link. https://osf.io/rkn7j/

Sample keys for each patient can be found at the following public link. https://osf.io/v7ehj/

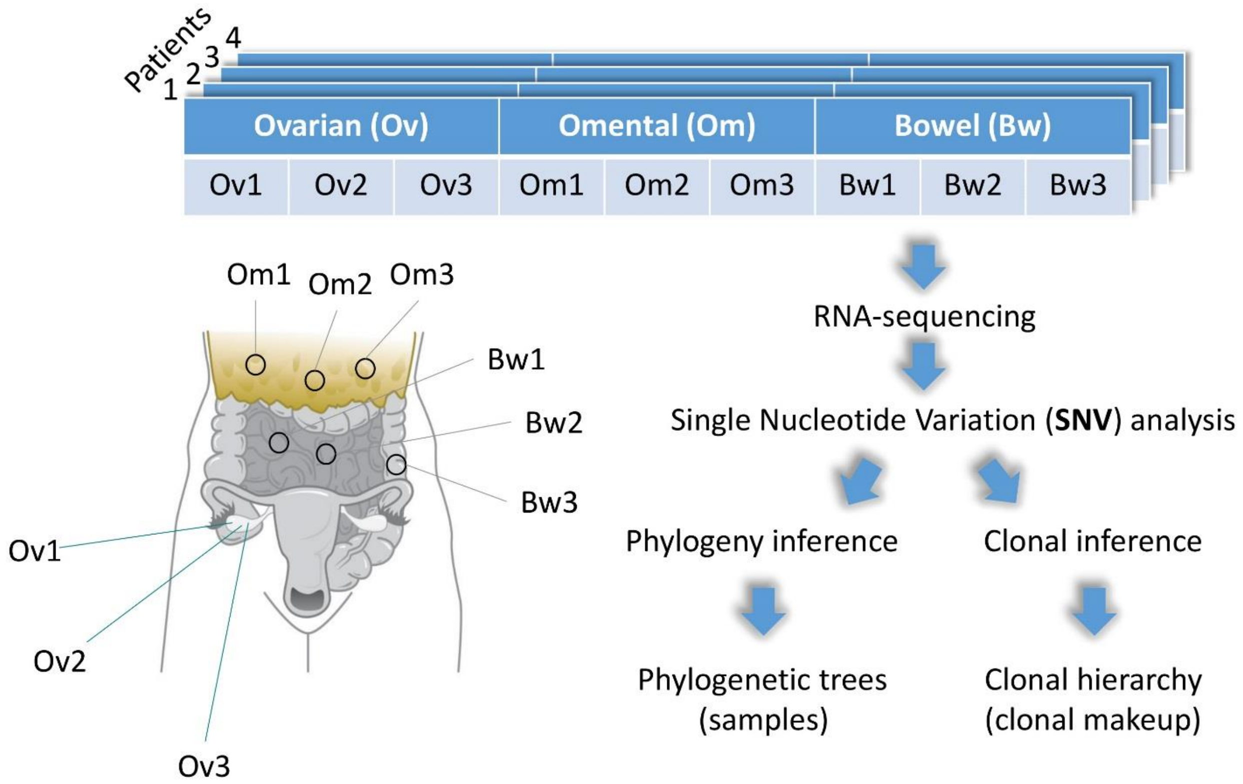

Fig. S1. *Study design*. Nine samples representing ovarian carcinomas, omental metastases, and bowel metastases were subjected to RNA sequencing, SNV analysis, phylogenetic tree reconstruction and clonal inference.

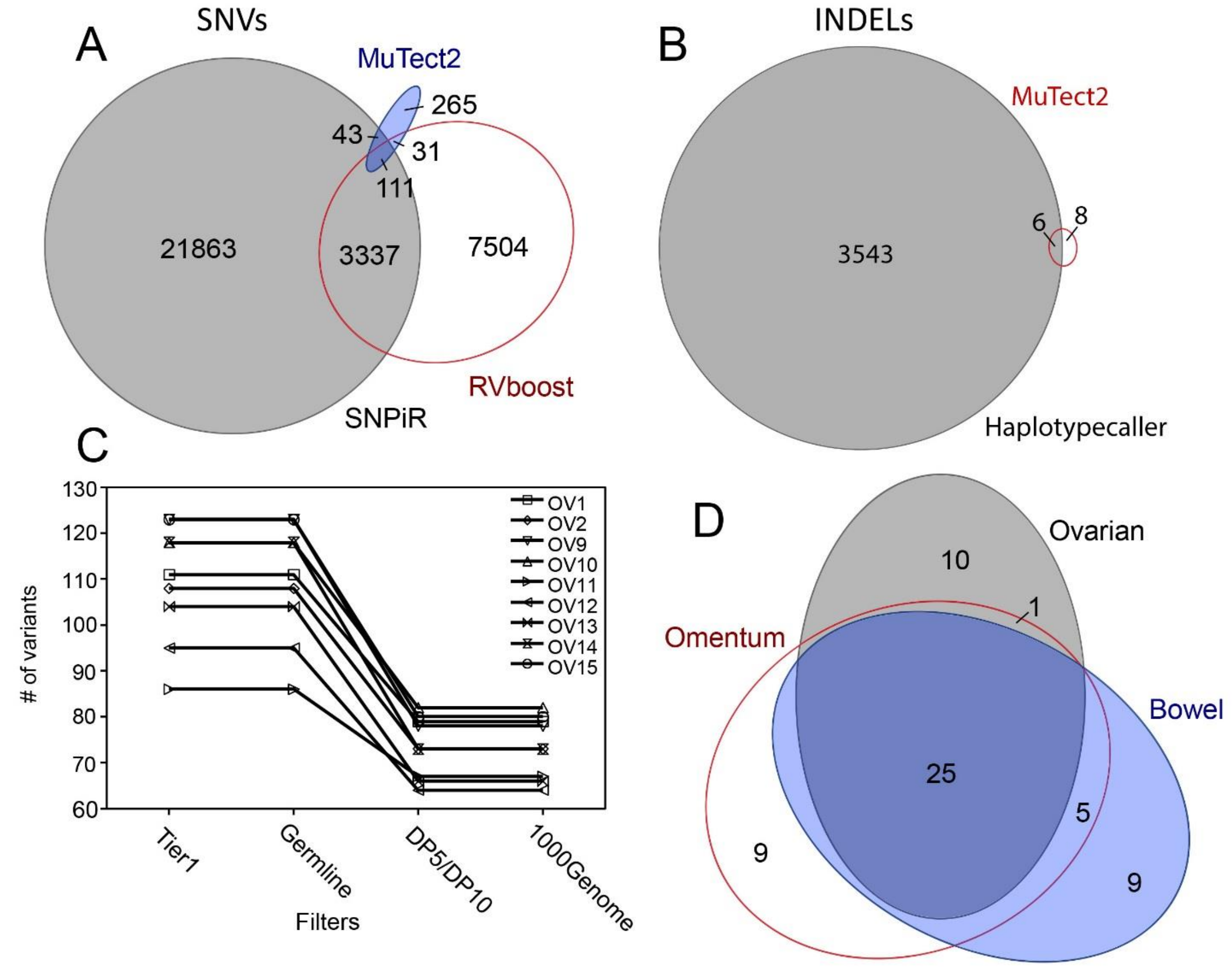

Fig. S2. *Consensus calling and filters of SNVs and INDELs*. (**A**) Venn diagram of SNVs discovered by three methods in one representative carcinoma sample. Note that the number of variants discovered by MuTect2 is very small because it performs joint calling using normal and tumor sequencing data sets and reports only somatic mutations. (**B**) Venn diagram of INDELs discovered by Haplotypecaller and MuTect2 in one representative carcinoma sample. (**C**) The number of somatic variants detected in nine carcinoma samples from one representative patient. Germline filter has no effect because MuTect2 performs joint calling between normal and carcinoma samples and reports somatic variants only. Coverage filter (read depth (**DP**) ≥ 5 for normal sample and DP ≥ 10 for tumor samples) has an effect on the number of somatic variants found in each carcinoma samples because variants with less than 10X coverage are filtered out. 1000 Genome population variant filter has no effect on the detected variants, suggesting that the likely germline variants are filtered out in prior steps. (**D**) Venn diagram of somatic variants (SNVs and INDELs) segregated by carcinomas from different anatomical sites.

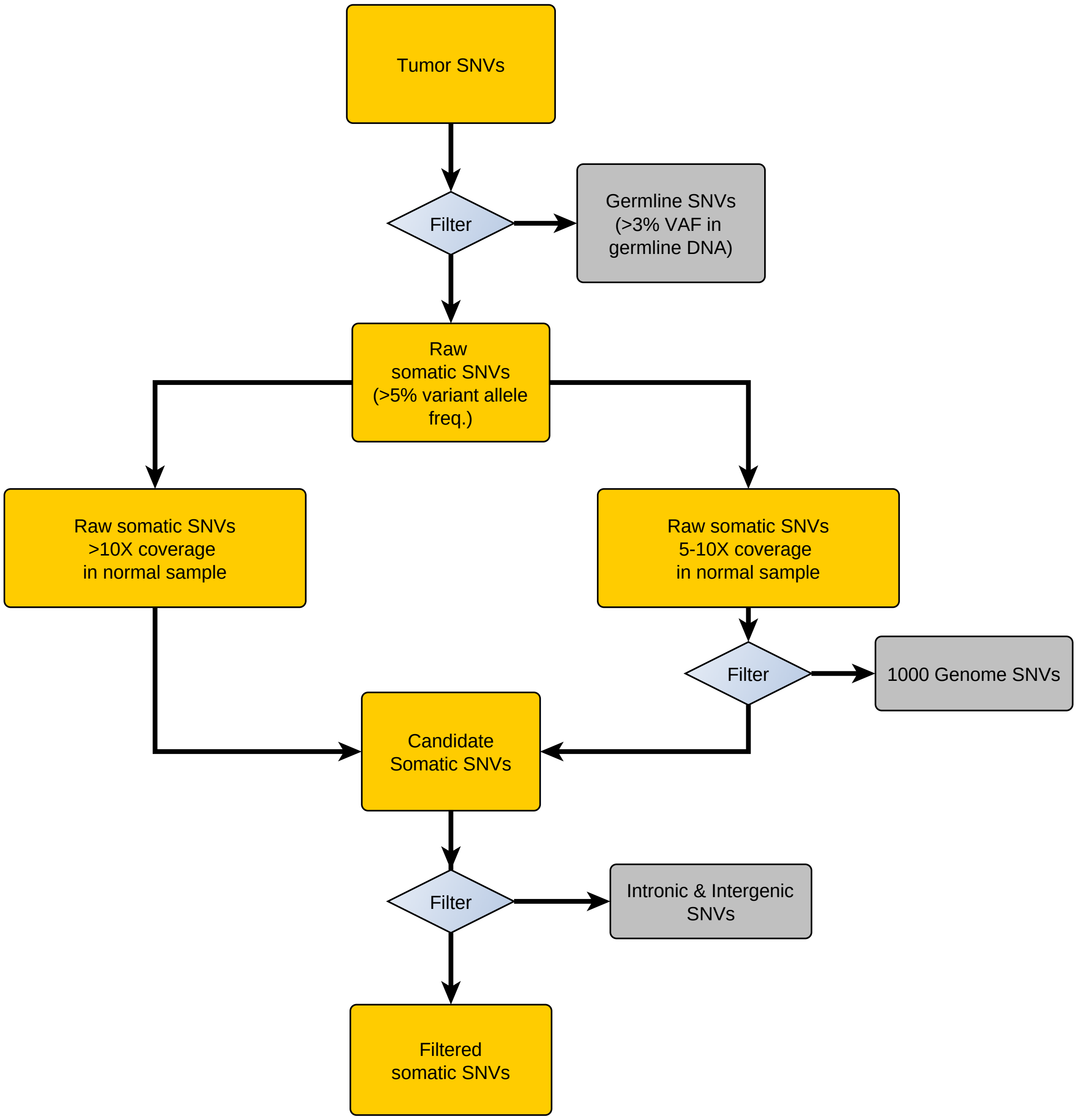

Fig. S3. The consensus tumor SNVs (from three independent calling methods) were subjected to additional filters. First, if the same variant is found at >3% frequency in germline DNA, these somatic variants are considered as germline variants and are filtered out. Second, we filtered out INDELs with <10% variant allele frequencies. Third, if variant position has >10X coverage in normal sample but no variant is found in normal sample, these somatic variants are considered as candidates. If variant position has 5-10X coverage in normal sample and if the 1000 Genome has reported the same variant, we considered these variants as germline variants and are filtered out. Finally, we filtered out intronic and intergenic variants to obtain final filtered somatic SNVs that are presented in Table 1. To get shared and unique SNVs, these variants are further filtered out so that we only consider variants at positions where we have read depth of ≥ 5X in normal DNA and ≥ 10X in **all** tumor RNA.

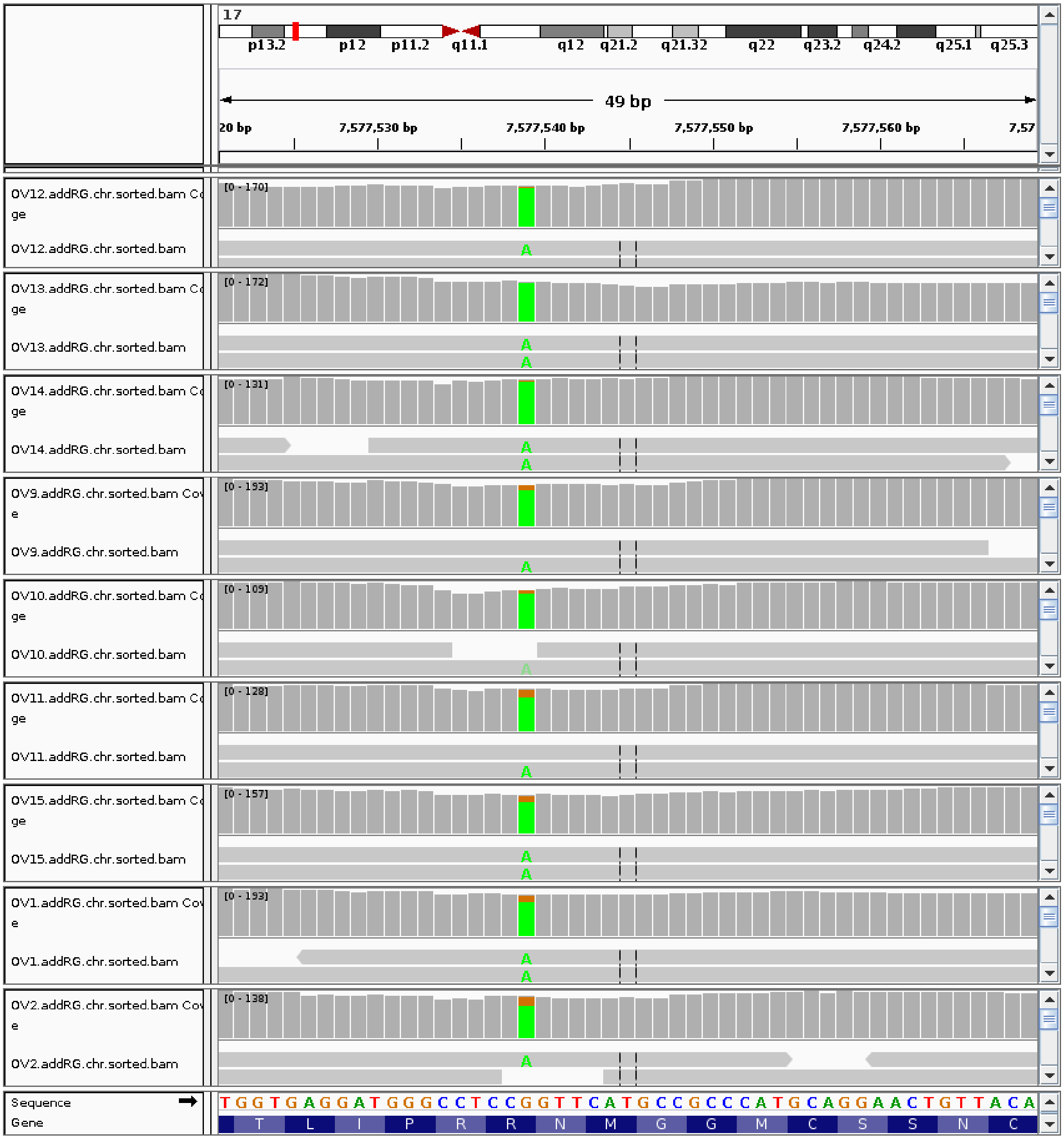

Fig. S4A. *TP53* mutation (R248W) in Patient 1 is observed at high allele fraction in all nine tumor samples (indicated by green fraction).

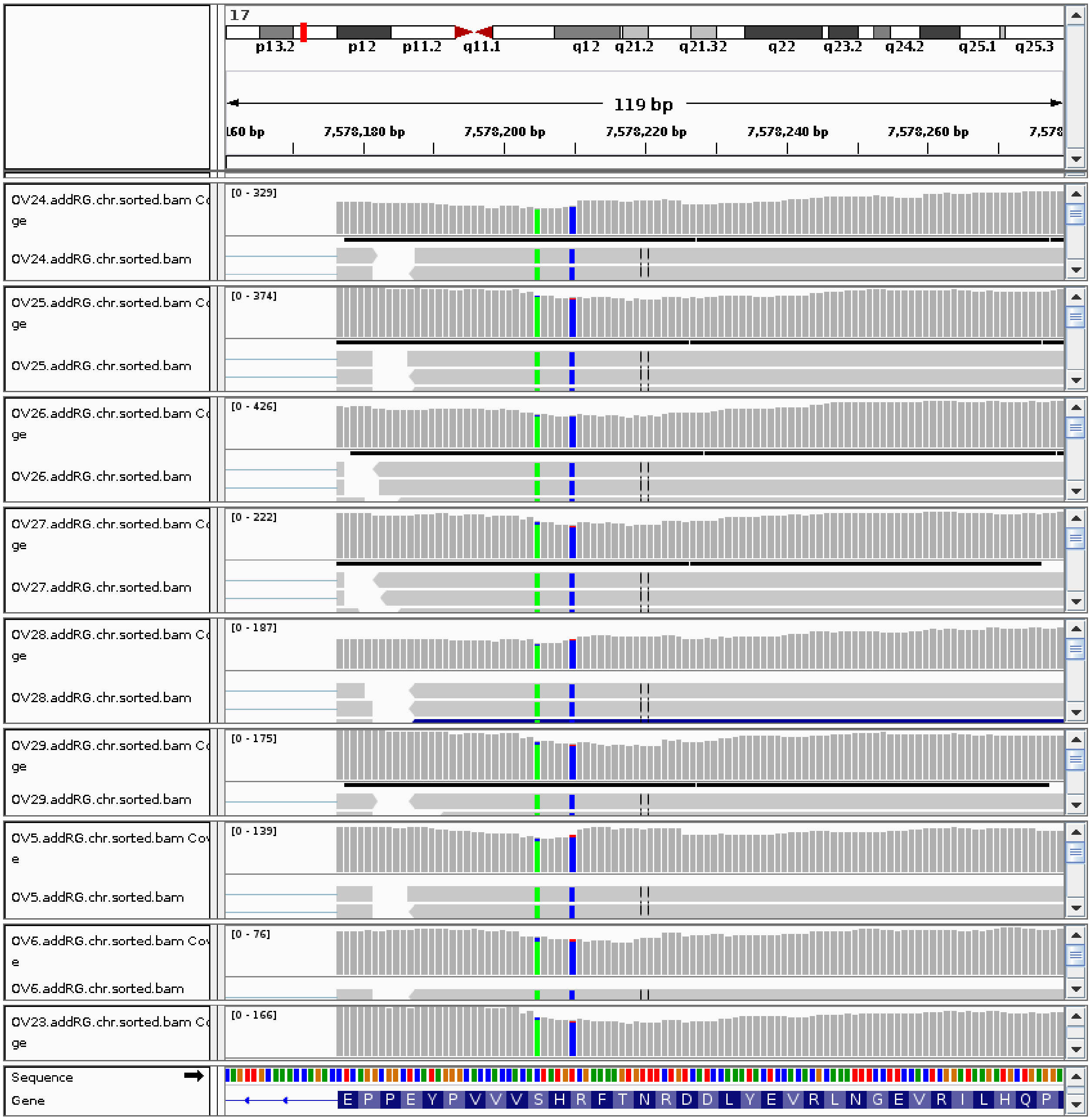

Fig. S4B. *TP53* mutation (S215I) in Patient 3 is observed at high allele fraction in all nine tumor samples (indicated by green fraction). Note that the codon that codes R213 has a germline SNP (CGT and CGG). This patient has the germline homozygyous CGG SNP (indicated by blue fraction in each sample).

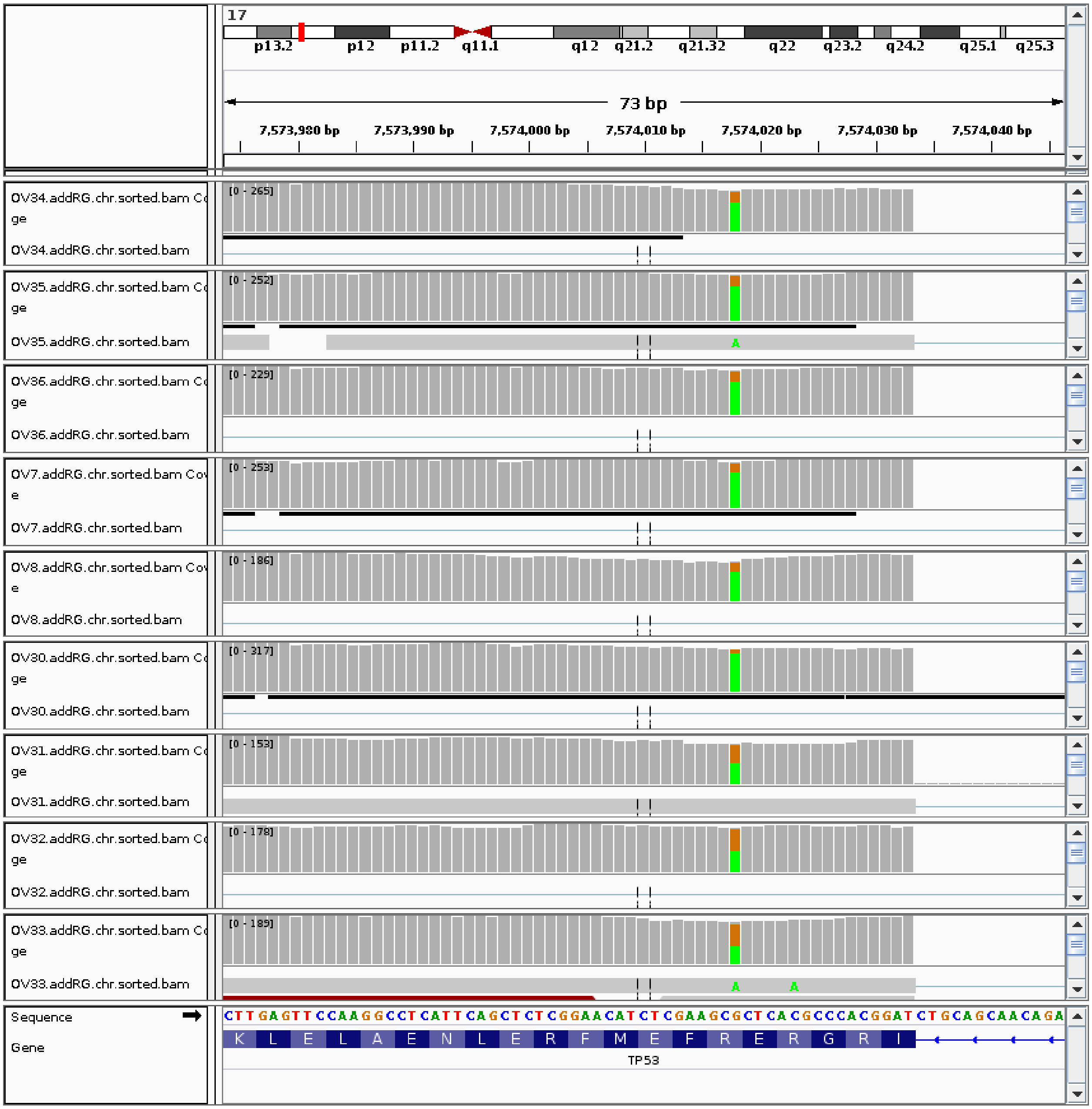

Fig. S4C. *TP53* mutation (R337C) in Patient 4 is observed at high allele fraction in some tumors (indicated by green fraction).

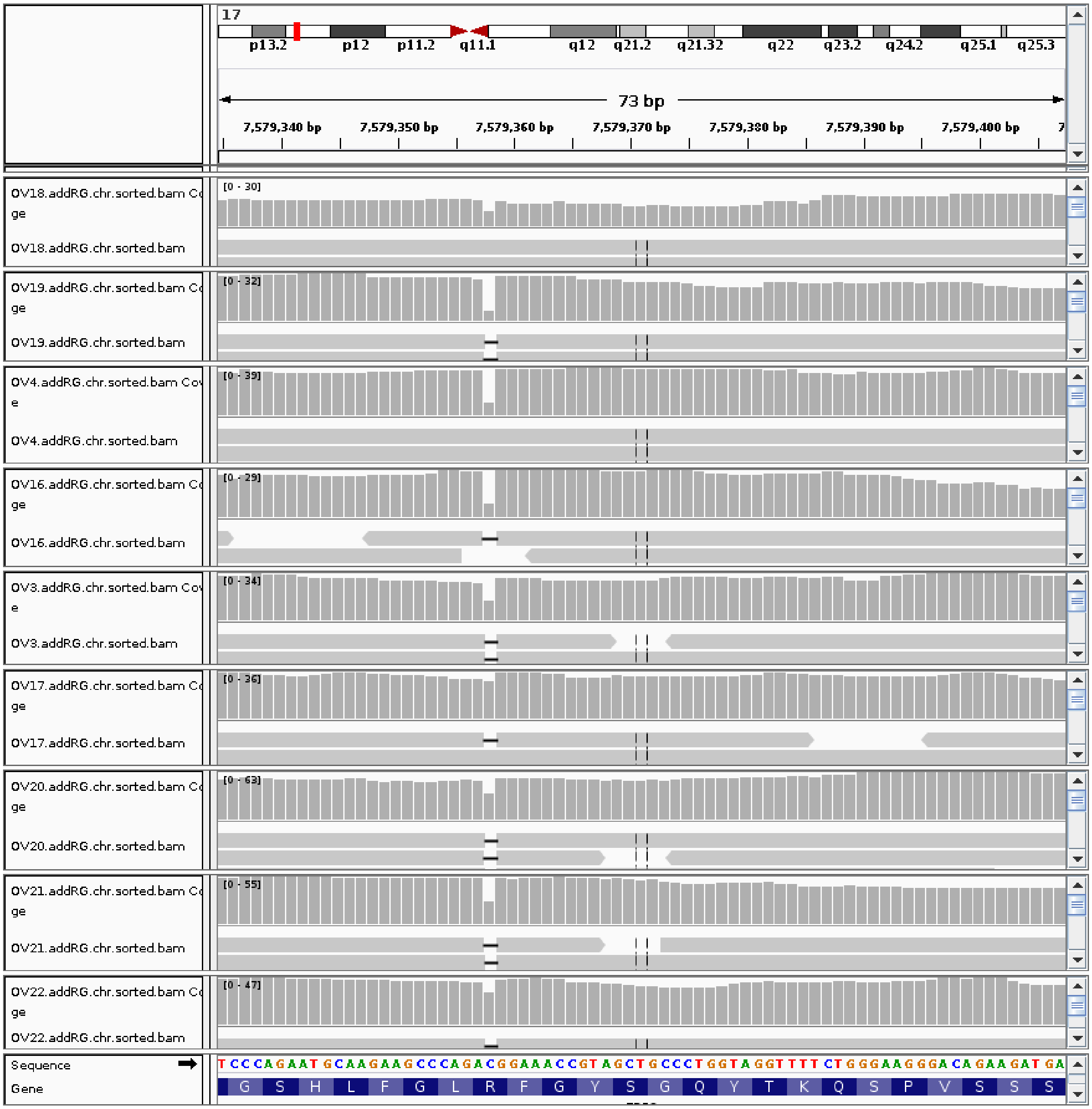

Fig. S4D. *TP53* mutation (R110fs*13) is observed in Patient 2. A single-base deletion is indicated by a gap in sequence and drop in coverage at the position. We observed lower overall coverage associated with *TP53* locus in all tumor samples in Patient 2 (<40X coverage of the *TP53* transcript in majority of tumor samples in this patient). In contrast, we obtained over 150 reads / base position mapping to *TP53* locus in almost all tumor samples in other Patients (Fig. S4A-C). These results indicate *TP53* gene expression is markedly decreased in Patient 2 compared to other patients. These results are consistent with the non-sense mediated decay of *TP53* gene transcript associated with frame-shift mutation observed in Patient 2.

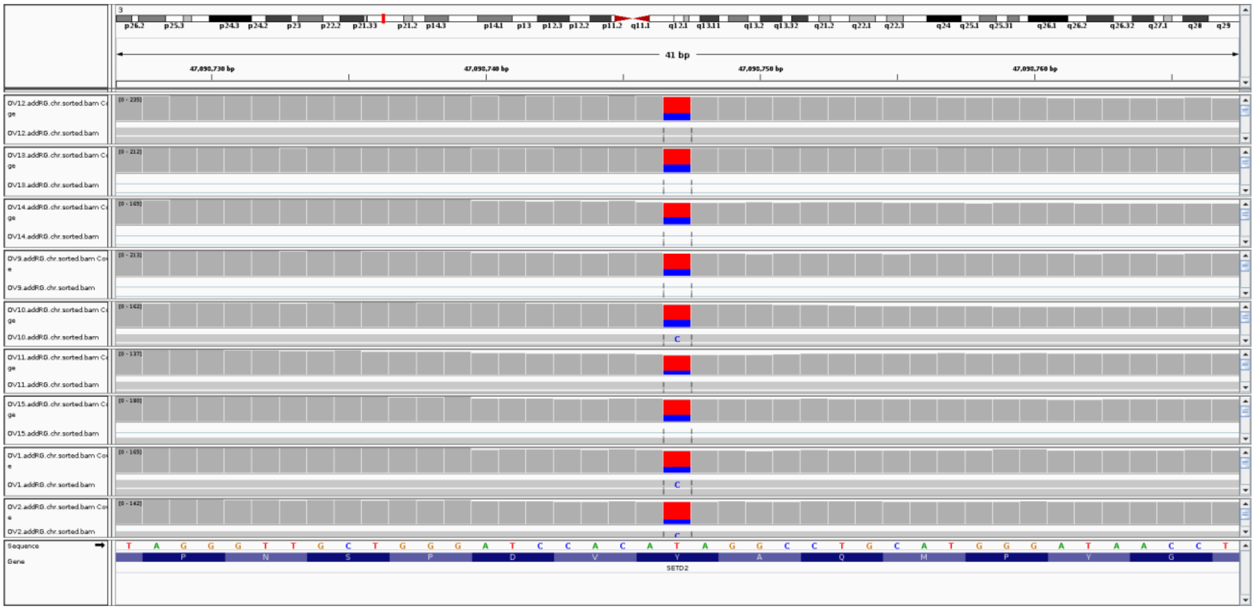

Fig. S5. *SETD2* mutation (Y2176C) in Patient 1.

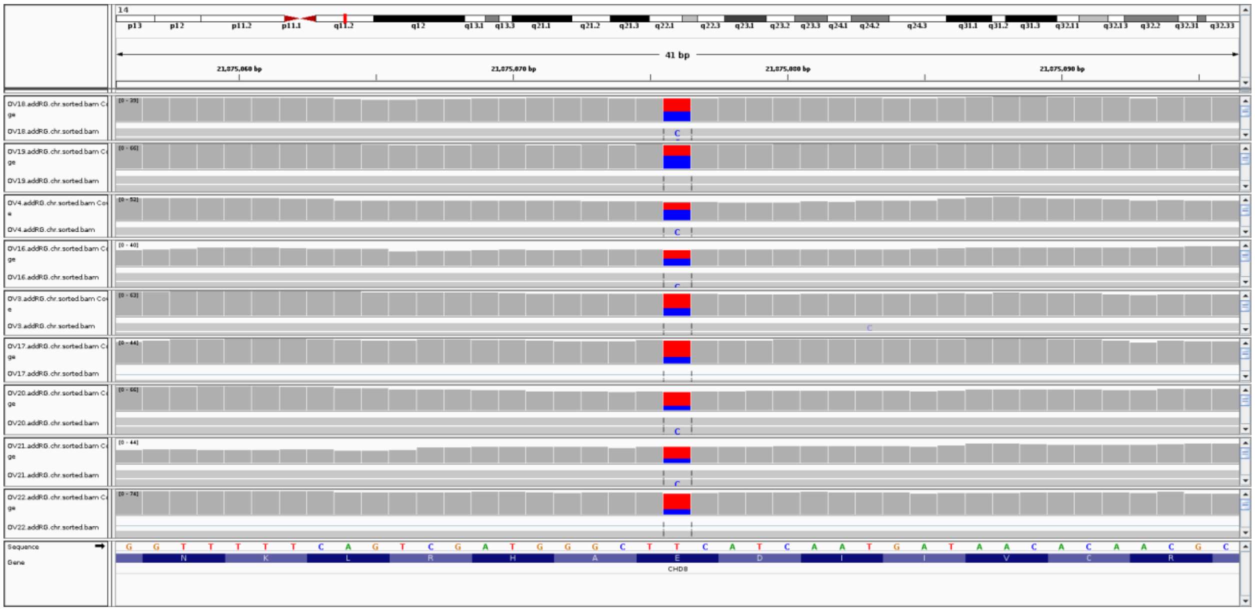

Fig. S6. *CHD8* mutation (E670G) in Patient 2.

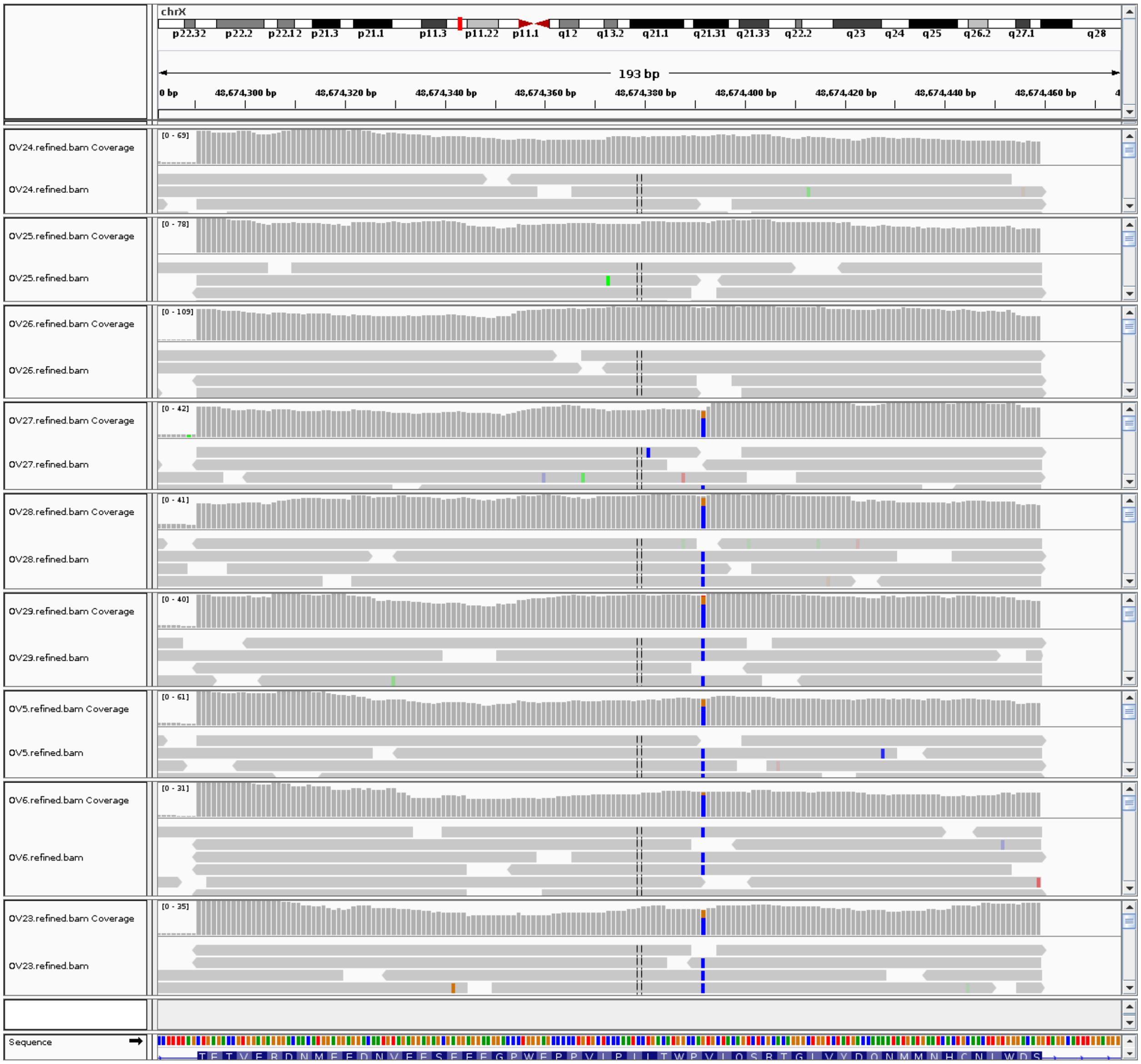

Fig. S7. *HDAC6* mutation (V476L) in Patient 3

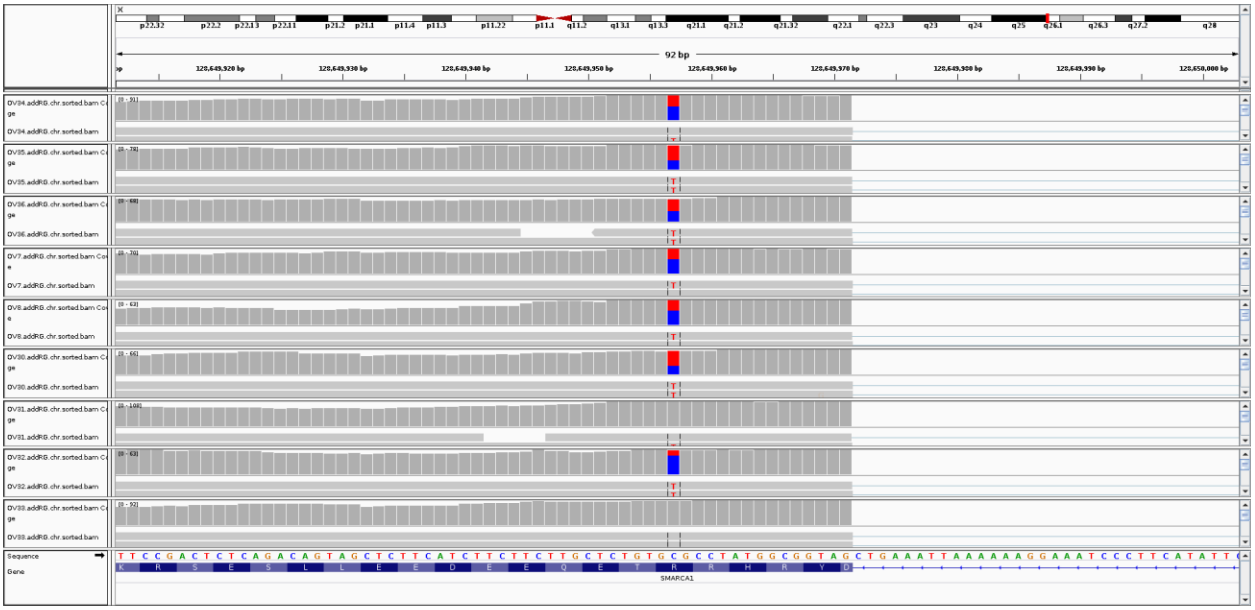

Fig. S8. *SMARCA1* mutation (R148H) in Patient 4.
